# Computational Evaluation of the Inhibition Efficacies of HIV Antivirals on SARS-CoV-2 (COVID-19) Protease and Identification of 3D Pharmacophore and Hit Compounds

**DOI:** 10.1155/2020/8818008

**Published:** 2020-09-21

**Authors:** Vinod P. Raphael, Shaju K. Shanmughan

**Affiliations:** Department of Chemistry, Government Engineering College, Thrissur, Kerala, 680009, India

## Abstract

Severe acute respiratory syndrome coronavirus 2 (SARS-CoV-2) is the novel coronavirus behind the fast-spreading coronavirus disease 2019 (COVID-19). Pharmaceutical researchers are currently researching medications or preventive vaccines that may be used to treat and combat the spread of COVID-19. Health practitioners all over the world are treating patients with currently available antiviral drugs, primarily the protease inhibitors used for HIV treatment. The present study mainly aims to evaluate the potencies of eight anti-HIV drugs to inhibit coronavirus protease using *in silico* methods. Derivation of pharmacophore, identification of hit molecules, and checking their virtual inhibition efficacies on the COVID-19 protease were also carried out in the present investigation. Classification of eight drug molecules (atazanavir, darunavir, fosamprenavir (amprenavir—metabolised product), saquinavir, lopinavir, ritonavir, nelfinavir, and indinavir) based on their molecular structures was completed and reported. The X-ray crystallographic structure of the main protease of coronavirus (SARS-CoV-2 protease) was obtained from the Protein Data Bank and prepared for computational studies using Edu PyMOL software. Docking studies were performed with AutoDock Vina software, and the results were evaluated with Discovery Studio software. The binding scores of the drugs on protease followed the order saquinavir > nelfinavir > lopinavir = indinavir > darunavir > amprenavir > ritonavir > atazanavir. Web servers such as PharmaGist and ZINCPharmer were employed to derive the 3D pharmacophore and to identify potential hit compounds, respectively. The identified hit molecules were docked with the SARS-CoV-2 protease and analysed. A detailed account of the type of interaction between the protease and the molecules is discussed. The majority of hit compounds displayed appreciable binding affinities on coronavirus protease. Three hit compounds possess structures similar to that of natural products, viz., flavonoids, and nucleoside. These molecules were hydrophilic and slightly deviated from Lipinski parameters. All other derived molecules obeyed the Lipinski rule. *In vitro*, *in vivo,* and toxicological studies of these compounds have to be performed before checking the actual druggability of these compounds.

## 1. Introduction

COVID-19 has become one of the most contagious and destructive viral diseases of the 21^st^ century. The rapidly spreading viral flu originated in China [1] and was identified in December 2019, following which it was confirmed that it is caused by a type of coronavirus, i.e., SARS-CoV-2 (severe acute respiratory syndrome coronavirus 2). On 11 March 2020, the World Health Organization declared that COVID-19 had become a global pandemic disease [2]. Many seriously affected countries resorted to complete population lockdown for several days/weeks to reduce the spread of the disease. The impact of lockdown was global and was disruptive to the world economy. The rate of spread of COVID-19 and the death rate due to respiratory illness and multiorgan damage are still not under control even after the implementation of lockdown. More than 23 million people have been affected, and 8 lakh people have lost their lives as of 23 August 2020 [3]. COVID-19-infected patients may have symptoms such as nasal congestion, sore throat, fever, and dry cough in early stages [4, 5]. Diarrhoea, shortness of breath, pneumonia, etc., may develop in later stages [5, 6].

Rigorous studies are being conducted by pharmaceutical scientists and medical practitioners all over the world to find out an effective medication or a preventive vaccine to control the spread of the new coronavirus. Since there is no specific drug discovered to cure COVID-19, doctors are treating the patients by the symptoms. Investigators say that it will take hardly 1-2 years to develop an effective formula against coronavirus. Many doctors treat their COVID-19 patients with available drugs in the market. Interferon alfa-2b [7, 8], developed by Cuba in 1981 to fight against dengue fever [9], is one of the antivirals used in China to fight COVID-19. Actemra [10, 11], an antibody medication (an anti-inflammatory drug for rheumatoid arthritis), is also used in China for the treatment of COVID-19 [12]. The United States has given permission to conduct clinical trials of the drug Kevzara (anti-inflammatory) [13, 14] in COVID-19 patients.

Remdesivir [15], hydroxychloroquine [16, 17], favipiravir [18], lopinavir [19], ritonavir [20], etc., are the common antiviral medications used for COVID-19. Among these, the first three are mainly used for diseases Ebola [21], influenza [22], and malaria [23], respectively. Lopinavir and ritonavir are anti-HIV drugs [24–27]. Some experienced medical practitioners believe that anti-HIV drugs that fall in the protease inhibitor class may be effective against coronavirus. They treat COVID-19 patients with anti-HIV protease inhibitor drugs such as lopinavir and ritonavir. The effectiveness of these drugs against COVID-19 may vary due to the capacity to bind strongly on the SARS-CoV-2 protease, which is the large molecule created by the coronavirus during their multiplication in the body.

In the present investigation, *in silico* evaluation of eight anti-HIV drugs (protease inhibitors) such as atazanavir, darunavir, fosamprenavir, saquinavir, lopinavir, ritonavir, nelfinavir, and indinavir was performed on the SARS-CoV-2 protease. Eight antivirals were classified into four types based on their molecular structures. To find similarly structured and more effective drugs against COVID-19, 3D pharmacophore derivation of these molecules was done. Molecules having identical pharmacophore features were chosen from the ZINC database, and computational docking studies were performed on coronavirus protease. The stability of protease-ligand complexes formed between the antivirals/hit molecules with the SARS-CoV-2 protease was well described based on the molecular characteristics of the compounds.

## 2. Materials and Methods

### 2.1. COVID-19 Protease

Structurally, coronavirus is an RNA virus that triggers the synthesis of polyproteins [28] when entered into the host cells. Polyproteins play a significant role in the multiplication of coronavirus. The behaviour of coronavirus in the host cell is more similar to that of an mRNA. In addition to the synthesis of polyproteins, protease molecules are created during the replication of coronavirus. The main protease of coronavirus which originated in Wuhan, China, is the SARS-CoV-2 protease. The principal duty of the protease molecules is to break the polyprotein chain into small molecules.

**Figure 1:**
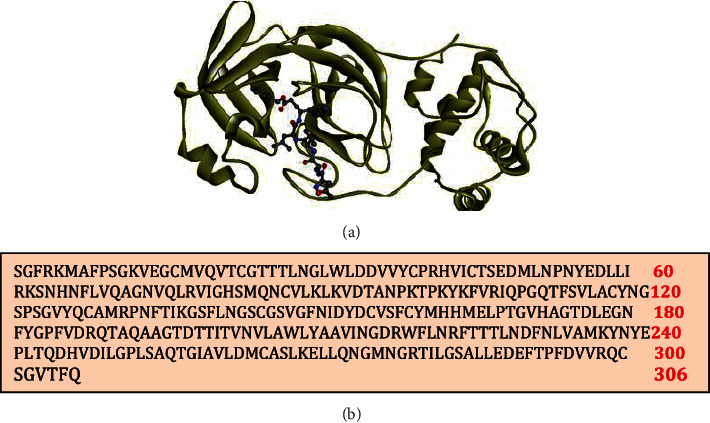
(a) Structure of the SARS-CoV-2 protease cocrystallised with the inhibitor N_3_. (b) Sequence of amino acids in the protease.

The COVID-19 main protease consists of 306 amino acids. The crystallographic structure of the protease was obtained from RCSB Protein Data Bank (PDB ID: 6lu7 (resolution: 2.6 Å; *R*-value free: 0.235)) [29].  Figure 1(a) represents the PDB structure of SARS CoV-2 protease cocrystallised with the inhibitor N_3_. The sequence of amino acids present in the protease is given in Figure 1(b).

### 2.2. Docking Studies

Edu PyMOL software (version 1.7.4) [30] was used for the preparation of the SARS-CoV-2 protease for docking studies. After removing the protein inhibitor and the crystallized water molecules, we performed the docking studies. After adding the H atoms, coordinates of the binding pocket of the receptor were determined by Discovery Studio v1.6.1.0.15355 software [31]. *In silico* virtual screening of the molecules on the SARS-CoV-2 protease was performed using AutoDock Vina software [32]. After the docking studies, we evaluated the nature of interactions of the molecules with the protease by Discovery Studio.

### 2.3. Derivation of Pharmacophore

Ligand-based 3D pharmacophore of drug molecules was derived using the PharmaGist web server. Out of the many results obtained, suitable pharmacophores were selected based on the scores awarded by the web server.

To find out active molecules other than anti-HIV drugs to inhibit the SARS-CoV-2 protease, we uploaded the features of 3D pharmacophores in the ZINCPharmer web server. Out of the various hit compounds displayed by the ZINC database, molecules having low RMSD values from the active sites of pharmacophore were chosen for docking studies.

In this work, we could not perform the molecular dynamic (MD) studies of protein-ligand complexes to validate the docking studies. Due to the COVID-19 outbreak, our educational institutions remain closed. We have little expertise in the area of MD simulation, and it will take a very long time to do the analysis. We hope that readers and researchers will not take this as a drawback of this manuscript. Apart from the docking studies, we have performed the 3D pharmacophore modelling and a clear-cut structure-based classification of anti-HIV drug molecules.

## 3. Results and Discussion

### 3.1. Classification of Anti-HIV Drugs

**Figure 2:**
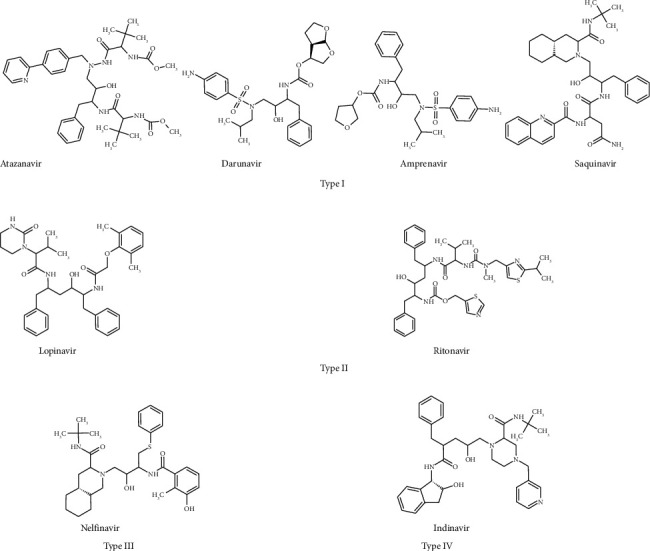
Molecular structures of eight HIV protease inhibitors.

Figure 2 shows the molecular structures of eight anti-HIV drugs. These HIV-antivirals can be divided into four types based on their backbone features. Atazanavir, darunavir, fosamprenavir, and saquinavir belong to Type I. The basic skeletal structures of these molecules consist of -CO-NH-CH(CH_2_-C_6_H_5_)-CH(OH)-CH_2_-N< linkage in which three carbon atoms occupy between two N atoms. In this type, fosamprenavir is a prodrug which metabolizes to amprenavir in the body. For computational evaluation, we took amprenavir as the drug molecule instead of fosamprenavir. Type II anti-HIV drugs include lopinavir and ritonavir, which have four carbon atoms trapped between two nitrogen atoms. The common skeletal moiety present in these molecules is -CO-NH-CH(CH_2_-C_6_H_5_)-CH_2_-CH(OH)-CH(CH_2_-C_6_H_5_)-N-CO-. Nelfinavir belongs to Type III. Even though the skeletal structure of this molecule bears three carbon atoms as in Type I, -CH_2_-S-C_6_H_5_ side chain is present instead of -CH_2_-C_6_H_5_. Indinavir belongs to Type IV since five carbon atoms are trapped between two nitrogen atoms. A slight structural modification of Type I molecules obtained by the interchange of -CO and NH groups present in the skeleton leads to the formation of Type IV.

### 3.2. Docking Studies of Anti-HIV Drugs on SARS-CoV-2 Protease

The docking score (binding energy) and the nature of the interaction of anti-HIV drugs with the amino acid residues of the SARS-CoV-2 protease are provided in Table 1. Out of the eight antivirals screened, saquinavir displayed the highest binding score (magnitude) with the COVID-19 protease (−9 kcal/mol). Saquinavir, lopinavir, nelfinavir, and indinavir showed >8 kcal/mol binding energy on the protease. The magnitude of binding scores of the drugs on the protease follows the order saquinavir > nelfinavir > lopinavir = indinavir > darunavir > amprenavir > ritonavir > atazanavir. The binding score of a ligand on a protein determines the stability of the protein-ligand complex. The number of strong hydrogen bonds and other interactions established between the ligand and amino acid residues of the binding pocket of the protein govern the binding score of the ligand. In general, the stability of the protein-ligand complex increases with the number of H-bonds for small molecules. However, for drug compounds with large molecular structures and high molecular masses, the stability of the protein-ligand adduct is greatly influenced by alkyl interactions and *π*-stacking interactions (hydrophobic) in addition to the conventional H-bonds [33, 34]. Since the molecular sizes of the anti-HIV drugs are not too low or too high, both H-bond interactions and hydrophobic interactions play a significant role in the receptor-ligand complex formation. In this article, we only discussed the 2D and 3D interaction plots of the COVID-19 protease-ligand complexes whose binding score (magnitude) is ≥8 kcal/mol.

#### 3.2.1. Type I Anti-HIV Drugs

The subsequent paragraphs contain the details of the interaction of anti-HIV drugs (atazanavir, darunavir, fosamprenavir, and saquinavir) with the SARS-CoV-2 protease. Atazanavir binds with the COVID-19 protease with three conventional H-bonds. This molecule showed a lower value (magnitude) of the binding score, i.e., 6.4 kcal/mol. Four *π* interactions were also established between the amino acid residues of the binding pocket and atazanavir. The binding affinity of darunavir was fair on the SARS-CoV-2 protease, and the molecule established three H-bonds and five *π* interactions with the receptor. Two unfavourable interactions between the molecule and the amino acid residues Ser144 and Thr25 of the protease may lead to the moderate binding affinity of darunavir. *In silico* evaluation of amprenavir (metabolised product of fosamprenavir) also displayed a fair binding score on the protease. The docking studies proved that three H-bonds, one *π*-stacking, and one alkyl interaction existed (not provided) in the protease-amprenavir complex.

**Figure 3:**
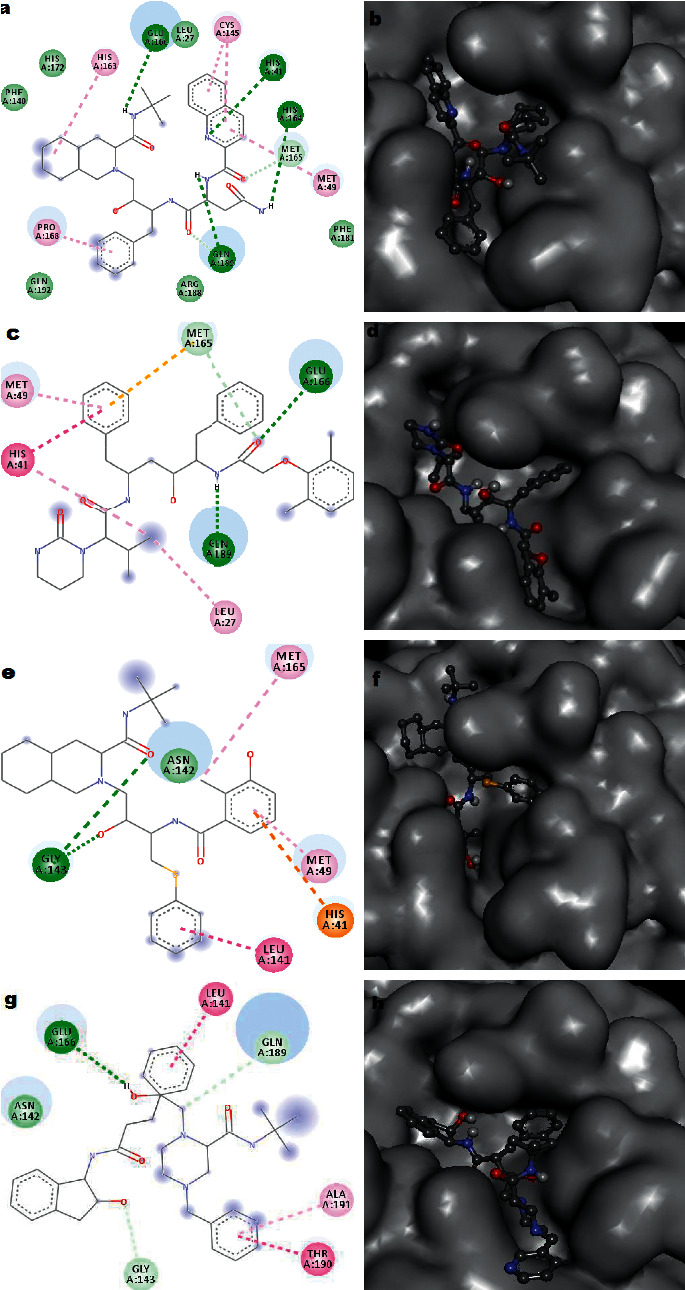
2D and 3D interaction plots of saquinavir (a and b), lopinavir (c and d), nelfinavir (e and f), and indinavir (g and h) with the SARS-CoV-2 protease.

The high affinity (−9.0 kcal/mol) of the anti-HIV drug saquinavir is due to the four-strong H-bond interactions and five *π*-R interactions with the receptor COVID-19 protease. Quinoline and benzene rings of saquinavir played significant roles in the receptor-ligand hydrophobic interactions. Out of the four H-bonds, three are originated (donor) from the saquinavir and one from the protease.  Figure 3(a) depicts the 2D interaction diagram of the COVID-19 protease with saquinavir. The 3D interaction plot (Figure 3(b)) portrays the most suitable pose of saquinavir molecule in the binding pocket of the COVID-19 protease.

#### 3.2.2. Type II Anti-HIV Drugs

Lopinavir and ritonavir belong to Type II anti-HIV drugs according to the structural classification. The COVID-19 protease-lopinavir complex consists of two conventional H-bonds and two *π*-R interactions. One *π*-stacking bond between the His41 ring and one of the benzene rings of the drug molecule can also be visualised from the structure of the complex. The lopinavir displayed a binding score of −8.1 kcal/mol on the SARS-CoV-2 protease. Figures 3(c) and 3(d) represent the 2D and 3D interaction plots of lopinavir with the main protease of SARS-CoV-2, respectively.

#### 3.2.3. Type III Anti-HIV Drug

Sulphur-containing nelfinavir belongs to Type III anti-HIV drug which interacts with the coronavirus protease with two strong acceptor H-bonds (ligand-O----H-N-Gly143-protease). Alkyl interaction with Met165, *π*-R interaction with Met49, and *π*-stacking bond with Leu141 also caused the formation of a robust receptor-nelfinavir complex having a binding energy of −8.2 kcal/mol. Figures 3(e) and 3(f) represent the 2D and 3D interaction plots of nelfinavir with the binding pocket of coronavirus protease, respectively.

#### 3.2.4. Type IV Anti-HIV Drug

Among the anti-HIV drugs considered for the present investigation, indinavir has obtained the maximum flexibility since the skeletal structure consists of five carbon atoms. According to virtual screening studies, this molecule is attached to the binding pocket of the COVID-19 protease firmly and displayed a score of −8.1 kcal/mol. Though the protein-ligand adduct consists of one conventional donor H-bond (ligand-O-H----Glu166), two strong *π*-stacking interactions with Leu141 and Thr190 and one *π*-R bond with Ala191 made the stable protease complex (Figure 3(g)).  Figure 3(h) shows the most favourable conformation of indinavir in the binding pocket of protease.

### 3.3. Derivation of 3D Pharmacophore

Ligand-based 3D pharmacophores of Type I anti-HIV drug molecules were derived using the PharmaGist web server. Based on the different conformations of the molecules, various pharmacophores were built by the web server in the decreasing order of the score. Out of different pharmacophores created, we chose the first two for further studies (J4-1 and J4-2). It was not possible to derive pharmacophores for Type II, III, and IV anti-HIV drugs since the server needs at least three similar molecules for the building of the same.

**Figure 4:**
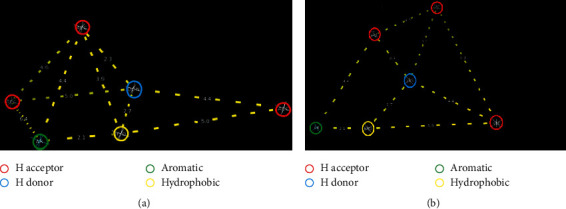
Structures of (a) J4-1 pharmacophore and (b) J4-2 pharmacophore.

The 3D pharmacophore (J4-1) of four anti-HIV drugs (Type 1) is given in Figure 4(a). It possessed a horse cart-like structure and consisted of three H-bond acceptor centres, one H-bond donor site, one aromatic ring, and one hydrophobic centre. Four common moieties present in the skeletal structure of the Type I molecules are participating in the creation of 3D pharmacophore, viz., the tertiary N atom (H-bond acceptor, right end of the pharmacophore), NH (H-bond donor), benzene ring (aromatic contributor), and the –CH_2_ group of the benzyl ring (hydrophobic behaviour). J4-2 pharmacophore acquired a camping tent-like structure. Four features, namely, aromatic, hydrophobic, H-donor, and one H-bond acceptor sites, are the same as that of the J4-1 structure. The distance between these common points was equal in both pharmacophore models. Two H-bond acceptor sites present in the J4-2 pharmacophore differed from that of the J4-1 pharmacophore skeleton. In general, J4-1 and J4-2 pharmacophores contain the main skeletal atom points present in Type 1 anti-HIV drugs.

#### 3.3.1. Docking Studies of J4-1 Group Molecules

**Figure 5:**
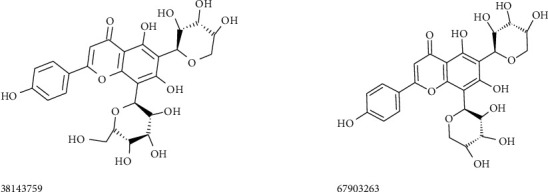
Structures of flavonoid molecules belong to J4-1 pharmacophore.

To find out more efficient molecules on the SARS-CoV-2 protease, the 3D pharmacophore features of antivirals were uploaded on the ZINCPharmer web server. Out of the various hit compounds displayed by the ZINC database, suitable molecules were selected based on the low RMSD values (<0.6). The 3D conformers of these hits were downloaded from the ZINC server and *in silico* studies were performed on the SARS-CoV-2 protease. ZINC IDs of molecules belong to the J4-1 pharmacophore, and their docking scores are listed in Table 2. Out of 10 hits chosen for the virtual screening studies, molecules having ≥8 kcal/mol (magnitude) binding affinity are listed in the table. The screened molecules displayed binding energies from −7.2 to −8.5 kcal/mol on the SARS-CoV-2 protease. Molecules having ZINC IDs 38143759 and 67903263 showed binding scores 8.5 and 8.4 kcal/mol, respectively (magnitude) on the SARS-CoV-2 protease. Among the J4-1 group, these molecules possessed high binding scores. The structures of these two molecules are provided in Figure 5. Both molecules possess a flavonoid structure and contain 9-10 hydroxyl groups. The first flavonoid of this group (38143759) interacted with the amino acid residue His41 of coronavirus protease using a strong hydrogen bond (2.4 Å). The second flavonoid of the J4-1 group (67903263) formed two H-bonds with the amino acid residue Asn142. Both molecules made one unfavourable interaction with the protease. Since these two molecules are the derivatives of natural products, the *in vivo* toxicity may be less. The effectiveness of these molecules to inhibit the SARS-CoV-2 protease and the toxicological studies have to be conducted and evaluated in the future.

#### 3.3.2. Docking Studies of J4-2 Group Molecules

**Figure 6:**
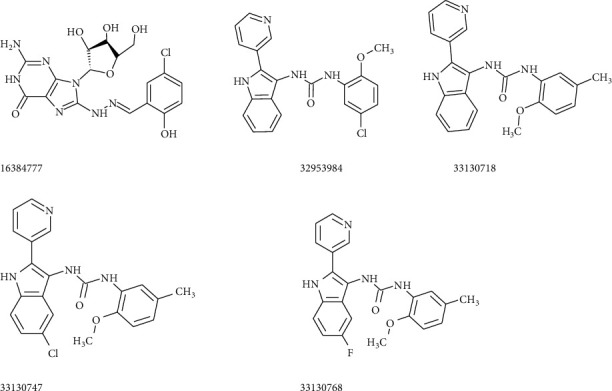
Structures of molecules belong to J4-2 pharmacophore.

The pharmacophore J4-2 was also uploaded in the ZINCPharmer website to get the most suitable molecules from the ZINC database. Eight molecules having low RMSD values (<0.6) were selected and screened for the binding affinity on the SARS-CoV-2 protease. These molecules showed binding energy values from −6.9 to −8.6 kcal/mol. Docking results of five molecules whose binding score (magnitude) >8 kcal/mol are given in Table 2. Structures of J4-2 group molecules are shown in Figure 6. The molecule having ZINC ID 16384777 owns a guanosine nucleoside unit and one aromatic ring. All other molecules are the derivatives of urea and have similar structures. One N atom of urea is bonded to a pyridine-substituted indole moiety, and the other N atom is attached to the benzene ring containing one methoxy unit. The molecule 33130768 showed the maximum binding affinity on the COVID-19 protease (8.6 kcal/mol). All molecules derived from urea bound to the COVID-19 protease using three conventional H-bonds with the amino acid residues Glu166, His164, and Gln189 present in the binding pocket. These molecules displayed *π*-S interaction with Met165 and/or Cys145 residue. Compared to the J4-1 group, all molecules of this group interacted with the protease with a large number of hydrophobic interactions. Presence of *π*-alkyl and *π*-stacking bonds was also established between the molecules and the amino acid residues of the COVID-19 protease. All urea-derived compounds did not exhibit any unfavourable interaction with the protease, but two negative interactions were seen between the amino acid residues Asn142 and Ser144 and the guanosine nucleoside molecule (16384777). Presence of one fluorine-Glu166 interaction was seen in the interaction plot of 33130768 on the SARS-CoV-2 protease (not given). Before claiming the druglikeness of these molecules, *in vitro* and *in vivo* studies have to be performed. Table 2 displays the types of interactions of these molecules on the SARS-CoV-2 protease.

### 3.4. Prediction of Druggability

The druglikeness of the molecules derived by the evaluation of ligand-based pharmacophores was checked using the Lipinski rule. The molecular masses of J4-1 group flavonoids were slightly higher than 500. According to the Lipinski rule, a most favoured mass of a drug-like molecule is 500 D. Majority of protease-inhibiting anti-HIV drugs have molecular mass >500 D and interact well with the binding pocket of the SARS-CoV-2 protease. It may be considered that the large-sized molecule is more fit in the binding pocket of the protease than a small one since the size of the binding site is high. The number of hydrogen bond donors (HBD) and hydrogen bond acceptors (HBA) was also slightly exceeded for 38143759 and 67903263 flavonoids as per the Lipinski rule. The negative values of logP indicate that both molecules are hydrophilic in nature. The polarisability of these molecules was comparable to that of a drug-like molecule predicted by the Lipinski rule. The nucleoside analogue molecule (ZINC ID 16384777, J4-2 group) also behaved as a hydrophilic compound. This molecule obeyed the Lipinski rule for the properties molecular mass, refractivity, and logP. HBD and HBA values were slightly deviated from the standard range. The remaining four molecules present in the J4-2 group well obeyed the Lipinski rule. These four molecules were lipophilic. This behaviour is essential for the drug metabolism since the absorption of orally consuming drugs takes place mainly through the walls of the intestine. The lipophilic nature of the molecules enhances the assimilation into the bloodstream. The details of the Lipinski parameters for the J4-1 and J4-2 group molecules are shown in Table 3.

## 4. Conclusions

Eight HIV protease inhibitor drugs were screened for their binding efficacy on COVID-19 main protease (SARS-CoV-2 protease) using computational docking studies. The binding scores of the drug molecules on the COVID-19 protease followed the order saquinavir > nelfinavir > lopinavir = indinavir > darunavir > amprenavir > ritonavir > atazanavir. Based on the skeletal structures, these drug molecules were classified into four types. Interaction of drug molecules with the binding pocket of the COVID-19 protease was evaluated based on the molecular structures. Saquinavir, nelfinavir, lopinavir, and indinavir showed a comparatively high binding score on the COVID-19 protease. Ligand-based 3D pharmacophores of the Type I molecules were derived, and structurally similar molecules were selected from the ZINC web server. Out of the various hit compounds obtained, flavonoid, nucleoside, and urea-based molecules displayed good binding scores on the COVID-19 protease. The structural behaviour, binding affinities, and drug-likeness of these molecules were determined and reported. We could not perform molecular dynamic studies of protease-ligand complexes due to the COVID-19 outbreak in India and the lack of authors' expertise. MD simulation studies for determining the stability of the complex and *in vitro*, *in vivo,* and clinical trials of these drugs and the molecules have to be performed for validating the real efficacy of coronavirus.

## Figures and Tables

**Table 1:** Binding score of anti-HIV drugs on the SARS-CoV-2 protease and types of interactions.

Anti-HIV drugs	Binding energy (kcal/mol)	Interactions	Amino acid residues
Atazanavir	−6.4	H-bond^*∗*^	Glu166-2(HB), Gly143(HB), Gly170(NC), Gln189-2(NC), Asn142(NC)
		*π* type^*∗∗*^	His41(*π*^+^), Cys145(*π*S), Cys145(*π*−*π*), Leu27(*π*-R)
		van der Waals	Leu167, His164, His163, Ser144, His172

Darunavir	−7.5	H-bond	His41(HB), Leu141(HB), Gly143(HB), His164(NC), Gln189(NC)
		*π* and R^#^ type	His41-3(*π*-R)Met49-2(R)
		van der Waals	Arg188, Cys145, His163
		Unfavourable	Ser144, Thr25

Amprenavir	−7.4	H-bond	Thr24(HB), His41(HB), Gly143(HB), Gly143(NC), His41(NC)
		*π* and R type	Leu141(*π*-stack), His41(*π*-S), Met165(R)
		van der Waals	Asn142

Saquinavir	−9.0	H-bond	Glu166(HB), His41(HB), His164(HB), Gln189(HB), Gln189(NC), Met165(NC)
		*π* and R type	Pro168(*π*-R), His163(*π*-R), Cys145-2(*π*-R), Met49(*π*-R)
		van der Waals	Phe140, His172, Leu27, Phe181, Arg188, Gln192

Lopinavir	−8.1	H-bond	Glu166(HB), Gln189(HB), Met165(NC)
		*π* and R type	His41(*π*-R), Met49(*π*-R), Met165(*π*-S), His41(*π*-stack), Leu27(R)
		van der Waals	Phe140,Cys145, His163, His164, His172

Ritonavir	−7.2	H-bond	Gly143(HB), Gln189(HB), Asn142(NC), Met165(NC), Glu166(NC), Glu189(NC), Thr190(NC), Thr26(NC),
		*π* and R type	Met49(*π*-R), Pro168(*π*-R), Met165(*π*-S), His41(*π*^+^), Glu166(*π*^−^), Thr25(*π*-*σ*), Met49(R)
		van der Waals	Leu27, Thr45, Ser46, Tyr54, Phe40, Cys145, Asp187, Arg188

Nelfinavir	−8.2	H-bond	Gly143-2(HB)
		*π* and R type	His41(*π*^+^), Leu141(*π*-stack), Met49(*π*-R), Met165(R)
		van der Waals	Asn142

Indinavir	−8.1	H-bond	Glu166(HB), Gln143(NC), Gln189(NC)
		*π* and R type	Leu141(*π*-stack), Thr190(*π*-stack), Ala191(*π*-R)
		van der Waals	Asn142

^*∗*^HB: conventional H-bond; NC: nonconventional H-bond. ^*∗∗*^*π*^+^ = *π*-cation, *π*^−^ = *π*-anion, *π*-R = *π*-alkyl, *π*-S = *π*-sulphur, and *π*-stack = *π*-stacking interactions. ^#^R = alkyl.

**Table 2:** Binding scores and nature of interactions of J4-1 and J4-2 group molecules on the SARS-CoV-2 protease.

Pharmacophore	ZINC ID	Binding energy (kcal/mol)	Interactions	Amino acid residues
J4-1	38143759	−8.5	H-bond^*∗*^	His41(HB), Thr26(NC)
			*π* type^*∗∗*^	Cys145(*π*-R), Met165-2(*π*-R)
			Unfavourable	Gly143(*π*-R)
	67903263	−8.4	H-bond^*∗*^	Asn142-2(HB), Thr26(NC), Leu141(NC)
			*π* type^*∗∗*^	Met165-2(*π*-R), Cys145(*π*-R)
			Unfavourable	Thr190

J4-2	16384777	−8.1	H-bond^*∗*^	Gly143(HB), Cys145(HB), Thr25(HB), Met165(NC)
			*π* type^*∗∗*^	His41(*π*-stack), Met49(*π*-R), Met49(R), Met165(*π*-S)
			Unfavourable	Asn142, Ser144
	32953984	−8.4	H-bond^*∗*^	Glu166(HB), His164(HB), Gln189(HB)
			*π* type^*∗∗*^	Met165(*π*-S), Leu167(R), Pro168(R), Met165(R), His41(*π*-stack), His41(*π*^+^), Cys145-2(*π*-S), Met49(*π*-R), Pro168(*π*-R), Met165(*π*-sigma)
	33130718	−8.3	H-bond^*∗*^	Gln189(HB), Gu166(HB), His164(HB)
			*π* type^*∗∗*^	Met165-2(*π*-S), Cys145-2(*π*-S), Pro168(*π*-R), His41(*π*-stack), His41(*π*^+^), Met49(*π*-R)
	33130747	−8.3	H-bond^*∗*^	Glu166(HB), His164(HB), Gln189(HB), Arg188(NC), Gln189(NC)
			*π* type^*∗∗*^	Met165(*π*-sigma), Met49(*π*-R),Cys145-2(*π*-S), Pro168(*π*-R)
	33130768	−8.6	H-bond^*∗*^	Glu166(HB), Gln189(HB), His164(HB)
			*π* type^*∗∗*^	Cys145-2(*π*-S), Met165(*π*-sigma), Met165(*π*-S), His41(*π*-stack), His41(*π*^+^), Pro168(*π*-R)
			Halogen(F)	Glu166(F)

**Table 3:** Lipinski parameters of J4-1 and J4-2 group molecules.

Property	38143759	67903263	16384777	32953984	33130718	33130747	33130768
M. mass	564	534	451	392.5	372	406.5	390
HBD	10	9	8	3	3	3	3
HBA	14	13	12	5	5	5	5
LogP	−1.91	−1.27	−1.01	5.54	5.19	5.84	5.33
M. ref	130.25	124.24	108.32	112.18	111.90	116.91	111.86

M. mass = molecular mass, HBD = hydrogen bond donor, HBA = hydrogen bond acceptor, P = partition coefficient, and M. ref = molar refractivity.

## Data Availability

All the data associated with this work are included in the manuscript.
